# Identification and localization of the structural proteins of anguillid herpesvirus 1

**DOI:** 10.1186/1297-9716-42-105

**Published:** 2011-10-05

**Authors:** Steven J van Beurden, Baptiste Leroy, Ruddy Wattiez, Olga LM Haenen, Sjef Boeren, Jacques JM Vervoort, Ben PH Peeters, Peter JM Rottier, Marc Y Engelsma, Alain F Vanderplasschen

**Affiliations:** 1 Central Veterinary Institute of Wageningen UR, P.O. Box 65, 8200 AB Lelystad, The Netherlands; 2 Virology Division, Department of Infectious Diseases and Immunology, Faculty of Veterinary Medicine, Utrecht University, P.O. Box 80.165, 3508 TD Utrecht, The Netherlands; 3 Proteomic and Microbiology (Pentagone), Interdisciplinary Center of Mass spectrometry (CISMa), University of Mons, Place du parc 20, B-7000 Mons, Belgium; 4 Laboratory of Biochemistry, Wageningen University, Dreijenlaan 3, 6703 HA Wageningen, The Netherlands; 5 Immunology-Vaccinology (B43b), Department of Infectious and Parasitic Diseases, Faculty of Veterinary Medicine, University of Liège, B-4000 Liège, Belgium

## Abstract

Many of the known fish herpesviruses have important aquaculture species as their natural host, and may cause serious disease and mortality. Anguillid herpesvirus 1 (AngHV-1) causes a hemorrhagic disease in European eel, *Anguilla anguilla*. Despite their importance, fundamental molecular knowledge on fish herpesviruses is still limited. In this study we describe the identification and localization of the structural proteins of AngHV-1. Purified virions were fractionated into a capsid-tegument and an envelope fraction, and premature capsids were isolated from infected cells. Proteins were extracted by different methods and identified by mass spectrometry. A total of 40 structural proteins were identified, of which 7 could be assigned to the capsid, 11 to the envelope, and 22 to the tegument. The identification and localization of these proteins allowed functional predictions. Our findings include the identification of the putative capsid triplex protein 1, the predominant tegument protein, and the major antigenic envelope proteins. Eighteen of the 40 AngHV-1 structural proteins had sequence homologues in related *Cyprinid herpesvirus 3 *(CyHV-3). Conservation of fish herpesvirus structural genes seemed to be high for the capsid proteins, limited for the tegument proteins, and low for the envelope proteins. The identification and localization of the structural proteins of AngHV-1 in this study adds to the fundamental knowledge of members of the *Alloherpesviridae *family, especially of the *Cyprinivirus *genus.

## Introduction

The *Alloherpesviridae *family, belonging to the *Herpesvirales *order comprises all bony fish and amphibian herpesviruses [1]. Currently, the family contains 4 genera with 11 species [2]. At least another 17 herpesviruses infecting bony fish have been described, but have not yet been sufficiently characterized to allow classification [1,3,4]. Many of these viruses cause serious disease and mortality in their respective host species, many of which are important aquaculture species. For example, channel catfish virus or *Ictalurid herpesvirus 1 *(IcHV-1) may cause up to 100% mortality in channel catfish (*Ictalurus punctatus*) fingerlings, which posed a significant problem in the big catfish aquaculture industry in the United States [5]. Koi herpesvirus or *Cyprinid herpesvirus 3 *(CyHV-3) is another highly contagious and virulent disease in its host species common carp and koi (*Cyprinus carpio *spp.), the first being one of the most economically valuable aquaculture species worldwide [6,7].

The eel herpesvirus anguillid herpesvirus 1 (AngHV-1) causes a hemorrhagic disease in the European eel, *Anguilla anguilla*, with increased mortality rates [8]. Because of its omnipresence in wild Western European eel stocks, AngHV-1 is regarded as one of the possible factors responsible for the decline of the wild European eel stocks since the 1980s [9]. Although the fundamental characteristics of herpesviruses of especially humans and mammals have been studied intensively, there is still little knowledge on the herpesviruses of lower vertebrates and invertebrates.

Despite their diversity in genes, host range and genome size, the virion structure is conserved throughout the entire *Herpesvirales *order [1]. Herpesvirus virions invariably consist of a large (diameter >100 nm) icosahedral nucleocapsid (*T *= 16) containing the genome, surrounded by a host-derived envelope with a diameter of about 200 nm, and an intervening proteinaceous layer called the tegument [10]. For a better understanding of the origins and replication cycle of members of the family *Alloherpesviridae*, the identification and characterization of the structural proteins of these alloherpesviruses is essential.

Mass spectrometry (MS) is a useful technique to identify proteins, particularly when sequence information about the protein composition is available [11]. The complete genome sequences of 5 alloherpesviruses have been determined to date and are publicly available: IcHV-1 [12], *Ranid herpesvirus 1 *(RaHV-1) and *Ranid herpesvirus 2 *(RaHV-2) [13], CyHV-3 [14] and AngHV-1 [15]. In 1995, Davison and Davison identified a total of 16 principal structural proteins for IcHV-1 by MS [16]. To enable assigning the identified proteins to the different compartments of the herpesvirus virion (i.e. capsid, tegument and envelope), complete virions were fractionated into a capsid-tegument and an envelope fraction, and premature capsids were isolated directly from infected cell nuclei. Using this approach, 4 capsid proteins, 4 envelope proteins, 5 tegument proteins and 5 tegument-associated proteins were detected for IcHV-1.

Recently, a total of 40 structural proteins were identified by MS in mature CyHV-3 particles [17]. This number resembles the total number of structural proteins reported for members of the *Herpersviridae *family [18-25]. It is likely that the number of structural proteins detected earlier for IcHV-1 is an underrepresentation of the actual number, caused by the limited sensitivity of MS at the time. The CyHV-3 structural proteins were assigned to the different herpesvirus compartments on the basis of sequence homology and bioinformatics [17]. Since sequence homology between CyHV-3 and IcHV-1 is limited, the putative location in the virion of the majority of the identified proteins could not be assigned.

The current study aimed at identifying and characterizing the structural proteins of AngHV-1. The approach in fact entails a combination of the capsid retrieval and virion fractionation techniques previously used for IcHV-1 [16], and the high sensitivity liquid chromatography tandem mass spectrometry (LC-MS/MS) approach used for CyHV-3 [17]. The envelope proteins were further characterized using bioinformatics. The results of this study not only provide insight into the protein composition of the mature extracellular AngHV-1 virions, but also give a first indication of the conservation of structural proteins within the *Alloherpesviridae *family.

## Materials and methods

### Production and purification of AngHV-1 virions

The Dutch AngHV-1 strain CVI500138 [26] was isolated and propagated in monolayers of eel kidney (EK-1) cells [27] in 150 cm^2 ^cell culture flasks infected at a multiplicity of infection of 0.1 as described previously [15]. Virions were purified from the culture medium using a previously described protocol with some modifications [28]. Three days post-infection, cell culture medium containing cell-released mature virions was collected and cleared from cell debris by centrifugation at 3 500 × *g *for 20 min at 4°C (Hermle Labortechnik Z400K, Wehingen, Germany). From here on virus was kept on ice. Virus was pelleted by ultracentrifugation at 22 000 rpm for 90 min at 4°C, with slow acceleration and slow deceleration (Beckman Coulter Optima L70K Ultracentrifuge with rotor SW28, Brea, CA, USA). The pellet was resuspended in 1 ml TNE buffer (50 mM Tris-HCl, 150 mM NaCl, 1 mM EDTA, pH = 7.5) by pipetting and vortexing. The virus suspension was layered onto a 10 to 60% linear sucrose gradient in TNE buffer. Following ultracentrifugation (rotor SW41Ti, 22 000 rpm for 18 h at 4°C), the virus band was collected. Subsequently, the virus was washed in 10 volumes of TNE buffer and concentrated by ultracentrifugation (rotor SW41Ti, 30 000 rpm for 3 h at 4°C). The virus pellet was resuspended in 200 μL TNE buffer and stored at -80°C until further use.

### Fractionation of AngHV-1

Lipid envelopes were released from the capsid-teguments by incubation with a nonionic detergent as described previously [16]. Briefly, an equal volume of solubilization buffer (50 mM Tris-HCl, 0.5 M NaCl, 20 mM EDTA, 2% (v/v) Nonidet P40) was added to the virus solution, incubated on ice for 15 min, and microcentrifuged at 25 000 × *g *for 5 min at 4°C (Eppendorf 5417R, Hamburg, Germany). Supernatant containing the envelopes was collected by pipetting. The capsid-tegument pellet was washed by vortexing in 100 μL of ice-cold 0.5x solubilization buffer followed by microcentrifugation at 25 000 × *g *for 5 min at 4°C. The supernatant was discarded by pipetting, 50 μL of cold TNE-buffer was added, and the pellet was resuspended by probe sonication (MSE, London, UK) for 10 s. The envelope fraction was clarified further by three subsequent microcentrifugation steps (25 000 × *g *for 5 min at 4°C), each time collecting the supernatant by decantation. The capsid-tegument and envelope fractions were stored at -80°C until further use.

### Purification of AngHV-1 capsids

AngHV-1 infected EK-1 cells were washed with PBS to remove complete virus particles. Cells from 4 150 cm^2 ^cell culture flasks were collected by scraping using a rubber policeman in 9 mL ice-cold TNE buffer. Cells were lysed by adding 1 mL of 10% (v/v) Triton X-100 in TNE (final concentration 1% (v/v) Triton X-100) and probe sonication on ice for three times 20 s. Debris was pelleted by ultracentrifugation (rotor SW41Ti, 10 000 rpm for 10 min at 4°C). Capsids were purified by sucrose cushion (40% in TNE) ultracentrifugation (20 000 rpm for 1 h at 4°C). The pellet was resuspended in 0.5 mL TNE and further purified by centrifugation on a linear 10-60% sucrose gradient in TNE for 1 h at 20 000 rpm. The two resulting bands, presumably containing capsids, were separately collected as an upper and a lower band, washed in TNE buffer and pelleted by ultracentrifugation (20 000 rpm for 1 h at 4°C). The supernatant was discarded, the capsid pellets resuspended in 200 μL TNE buffer and stored at -80°C until further use.

### Electron microscopy

Nickel grids (400-mesh) with a carbon-coated collodion film were placed upside down on a drop of complete virion suspension, virion fraction suspension or capsid suspension, and incubated for 10 min. After incubation, grids were washed with distilled water and stained with 2% phosphotungstic acid (pH = 6.8). Grids were examined with a Philips CM10 transmission electron microscope (Amsterdam, The Netherlands).

### SDS-PAGE

Proteins in purified virions, virion fractions and capsids were analyzed by sodium dodecyl sulfate polyacrylamide gel electrophoresis (SDS-PAGE). Virions in TNE buffer were mixed 1 : 1 with denaturizing sample buffer containing dithiothreitol (DTT) and heated for 5 min at 95°C. Samples were loaded onto 12% NuPAGE Novex Bis-Tris gels (Invitrogen by Life Technologies, Carlsbad, CA, USA) and ran for 2 h at 80 V in NuPAGE MOPS SDS-running buffer (Invitrogen). Gels were stained with Coomassie blue R-250 (Merck, Whitehouse Station, NJ, USA) or Silver (PlusOne Silver Staining Kit, GE Healthcare, Chalfont St. Giles, UK).

### LC-MS/MS approach

The capsid proteins in the upper band were analyzed by SDS-PAGE and stained with Coomassie blue. The five visible protein bands were collected separately in gel slices. The gel segments were incubated in 10 mM DTT in 50 mM ammonium bicarbonate (ABC) buffer at 60°C for 1 h to reduce disulfide bridges and subsequently in 100 mM iodoacetamide (Sigma-Aldrich, St. Louis, MO, USA) in ABC buffer at room temperature for 1 h in the dark. After a final wash step with ABC buffer, the gel material was dried. Trypsin digestion was performed as described previously by Ince et al. [29]. In short, in-gel protein digestion was performed using sequencing grade modified porcine trypsin (Promega, Madison, WI, USA) in ABC buffer (10 ng/μL). After incubation overnight, samples were bath sonicated, and after centrifugation the basic supernatants were collected. The remaining gel pieces were extracted with 10% triflouroacetic acid (TFA), followed by 5% TFA, followed by 15% acetonitrile/1% TFA. The latter extracts were combined with the supernatants of the original digests, vacuum-dried, and dissolved in 20 μL 0.1% formic acid in water. The peptides resulting from this digestion were analyzed by LC-MS/MS as described previously [29].

### 1D gel/nanoLC-MS/MS approach

Proteins from purified virions and from the three virion fractions (capsid, envelope and capsid-tegument) were separated by SDS-PAGE on 4-20% acrylamide 7 cm gels (Invitrogen) and stained with Coomassie blue. Separated proteins in the gel were excised in 20 and 30 serial slices along the lane, for complete virions and virion fractions, respectively. Gel slices were submitted to in-gel digestion with sequencing grade modified trypsin as described previously [17]. Briefly, gels were washed successively with ABC buffer and ABC buffer/acetonitrile (ACN) 50% (v/v). Proteins were reduced and alkylated using DTT and iodoacetamide followed by washing with ABC and ABC/ACN. Resulting peptides were analyzed by 1D gel/nanoLC-MS/MS using a 40 min ACN gradient as described by Mastroleo et al. [30].

### 2D nanoLC-MS/MS approach

Only proteins of purified complete virions were submitted to 2D nanoLC-MS/MS analysis. Proteins were extracted from complete virions using guanidine chloride (GC) as described previously [17]. In short, the virions were suspended in 6 M GC and sonicated for 5 min and shaken at 900 rpm for 30 min at room temperature. After centrifugation the proteins were reduced with 10 mM DTT at 60°C for 30 min and alkylated with 25 mM iodoacetamide at 25°C for 30 min in the dark. Proteins were recovered by acetone precipitation and dissolved in 50 mM Tris/HCl (pH = 8), 2 M urea. The proteins were digested overnight at 37°C with trypsin (enzyme : substrate ratio = 1 : 50). Tryptic peptides were cleaned using spin tips (Thermo Fisher Scientific, Waltham, MA, USA) according to the manufacturer's instructions. Proteins were analyzed by 2D (strong cation exchange, reverse-phase) chromatography and online MS/MS, as described by Mastroleo et al. [30] except that only 3 salt plugs of 25, 100 and 800 mM NH_4_Cl were analyzed in addition to the SCX flow through.

### MS/MS analyses

Peptides were analyzed using an HCT ultra ion Trap (Bruker, Billerica, MA, USA). Peptide fragment mass spectra were acquired in data-dependent AutoMS(2) mode for 4 most abundant precursor ions in all MS scan. After acquisition of 2 spectra, precursors were actively excluded within a 2 min window, and all singly charged ions were excluded. Data were processed using Mascot Distiller with default parameters. An in-house Mascot 2.2 server (Matrix Science, London, UK) was used for dataset searching against the NCBI *Alloherpesviridae *database. The default search parameters used were the following: Enzyme = Trypsin; Maximum missed cleavages = 2; Fixed modifications = Carbamidomethyl (C); Variable modifications = Oxidation (M); Peptide tolerance ± 1.5 Dalton (Da); MS/MS tolerance ± 0.5 Da; Peptide charge = 2+ and 3+; Instrument = ESI-TRAP. Only sequences identified with a Mascot Score greater than 30 were considered, which indicates identity or extensive homology (*p*-value < 0.05). Single peptide identification was systematically evaluated manually. The exponentially modified protein abundance index (emPAI) [31] was calculated to estimate protein relative abundance for the complete virion extracts. The protein abundance index (PAI) is defined as the number of observed peptides divided by the number of observable peptides per protein. The exponentially modified PAI (10^PAI ^- 1) is proportional to protein content in a protein mixture in LC-MS/MS experiments.

### Bioinformatics

The amino acid sequences of all identified AngHV-1 structural proteins were analyzed using bioinformatic tools from the CBS website [32] to identify potential transmembrane domains (TMHMM) [33], signal peptides (SignalP) [34], and glycosylation sites (NetNGlyc [35] and NetOGlyc [36]).

## Results and Discussion

### Electron microscopy

Purified virus, fractionated virus, and purified capsids were checked for quality by transmission electron microscopy (EM, pictures not shown). The preparation of purified virus contained complete virions with intact or disrupted envelope, capsids and envelopes. The upper band of sucrose gradient purified capsids primarily contained capsids with an electron-lucent inner appearance, while the lower band showed capsids with an electron-dense core. No cell debris was seen in the capsid fractions. In the capsid-tegument fraction, only capsids and no envelopes were visible. The envelopes in the envelope fraction were largely unrecognizable and present as clusters of membrane remnants. Only very few capsids contaminated the envelope fraction. Overall, the virus and capsid purification as well as the virus fractionation could be considered successful, at least as evaluated by EM.

Davison and Davison [16] already mentioned for IcHV-1 that the resulting upper and lower bands after capsid purification parallel the density separation of mammalian herpesvirus capsids into A, B and C forms (in order of increasing density) as initially described by Gibson and Roizman [37]. The lower band consists of capsids comparable to the mature DNA-containing C form capsids, while the upper band comprises both the immature DNA-lacking B form capsids (containing additional core proteins) and the erroneous DNA-lacking A form capsids. For the sake of clarity in this paper we will follow the nomenclature as initially proposed for the IcHV-1 capsids: U capsids for the capsids found in the upper (U) band and L capsids for the capsids found in the lower (L) band [16]. The L capsid fraction contained significantly more DNA-containing capsids than the U capsid fraction (data not shown). This was in agreement with earlier observations on DNA content of premature mammalian herpesvirus capsids [38,39], and with the current view of herpesvirus capsids being first assembled around a scaffold with the DNA being inserted later [40].

### SDS-PAGE

**Figure 1:**
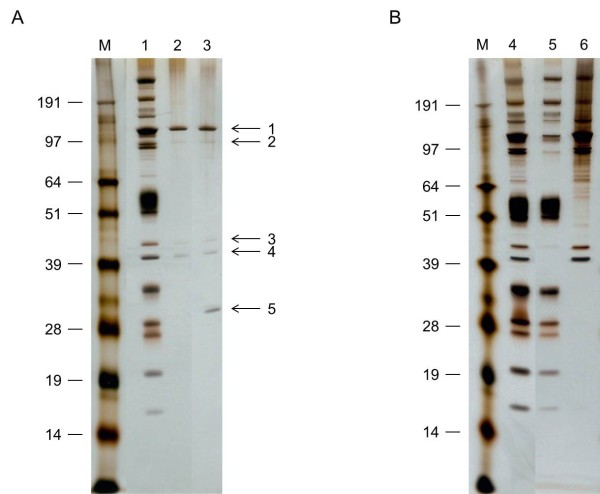
**One dimensional SDS-PAGE profile of different AngHV-1 virion fractions**. Loaded onto 12% Bis-Tris polyacrylamide and silver stained: 1a) proteins in the purified complete virions (lane 1), L capsids (lane 2) and U capsids (lane 3), numbered arrows indicate the excised protein bands which were analyzed by LC-MS/MS (Table 1); 1b) proteins in the purified complete virions (lane 4), envelope fraction (lane 5), and capsid-tegument fraction (lane 6). Molecular masses (kDa) are indicated on the left.

Proteins in purified virions, capsids and virion fractions were analyzed by SDS-PAGE to check purity and analyze the protein content of the different virion compartments. Thirty-five bands were visible for the complete virions using silver staining (Figure 1). Purified L capsids showed 4 clear bands, the U capsids showed an additional fifth protein band of low molecular weight not present in complete virus particles (Figure 1a). The envelope fraction resulted in 20 proteins (Figure 1b). The capsid-tegument fraction resulted in a smear in the high molecular weight region and at least 27 individual proteins could be differentiated. All four L capsid proteins were also present in the capsid-tegument fraction. Several proteins were predominant in either the envelope-tegument or the capsid-tegument fraction, but at least 8 proteins were clearly present in both fractions, presumably representing tegument-associated proteins.

### AngHV-1 capsid proteins

The five U capsid proteins were excised from a Coomassie blue stained gel and identified by LC-MS/MS. The proteins were identified as the major capsid protein (ORF104), the proteins encoded by ORF48 and ORF42, and the capsid triplex protein 2 (ORF36) (Table 1). The fifth protein, which did not seem to be present in the L capsids, appeared to be the capsid protease-and-scaffolding protein (ORF57). Indeed, in mammalian herpesviruses, this protein serves as a scaffold around which the capsid is built, and is proteolytically cleaved and extruded at the moment of DNA incorporation in the capsid [40].

**Table 1:** Capsid proteins of AngHV-1 as identified by 1D gel/nanoLC-MS/MS in purified U capsids

**ORF**^**a**^	NCBI ID	**Band**^**b**^	**Description**^**c**^	Predicted molecular mass (kDa)	**Number of peptides**^**d**^	Mascot score	**CyHV-3**^**e**^	IcHV-1	RaHV-1	RaHV-2
36	282174073	4	Capsid triplex protein 2	40.2	91	3724	ORF72	*ORF27*	ORF95	ORF131
42	282174079	3	*Capsid triplex protein 1*	42.9	75	3288	ORF66			
48	282174085	2		102.6	52	2935				
57	282174094	5	Capsid protease-and-scaffolding protein	78.0	114	5037	ORF78	ORF28	ORF63	ORF88
100	282174137	-		61.1	9	381	ORF90	ORF37	*ORF52*	*ORF78*
104	282174141	1	Major capsid protein	139.9	537	26277	ORF92	ORF39	ORF54	ORF80
126	282174163	-		22.4	2	89				

^a^the ORF are ordered by number, ^b^band number refers to the capsid protein bands shown in Figure 1a, ^c^properties newly suggested in this paper are presented in italics, ^d^number of peptides and Mascot score in the U capsid fraction, and ^e^homologous ORF in CyHV-3, IcHV-1, RaHV-1 and RaHV-2 are given, in case of marginal sequence identity (E-value > 10^-5 ^in a BLAST search limited to members of the *Alloherpesviridae *family) presented in italics.

Based on size and relative abundance compared to the capsid triplex protein 2 and the major capsid protein, AngHV-1 ORF42 is the best candidate to encode the capsid triplex protein 1 (expected ratio 1 : 2 : 3 [37,41]). ORF42 shows no convincing sequence homology with its putative functional homologue in IcHV-1 ORF53 [16]. It shows sequence homology, however, with ORF66 of the more closely related CyHV-3, encoding an abundant structural protein [17]. This low sequence conservation of the capsid triplex protein 1 is comparable with the sequence conservation among the capsid proteins of members of the *Herpesviridae *family [16]. Another highly abundant and large capsid protein is encoded by AngHV-1 ORF48, for which sequence homology was absent, however.

To obtain complementary data another gel loaded with U capsid proteins separated by SDS-PAGE was divided into 30 serial slices which were analyzed with 1D gel/nanoLC-MS/MS. This analysis resulted in the identification of another 6 low abundant proteins, of which 4 were contaminating other high abundant structural proteins, and 2 were additional putative capsid proteins. For the protein encoded by the spliced ORF100, 9 peptides were found in the U capsid fraction. Its high conservation among other alloherpesviruses hints to a possibly important but yet unknown function. The protein encoded by AngHV-1 ORF126 was little abundant (only 2 peptides in the U capsid fraction) and is not conserved in other herpesviruses. The overall high rate of capsid protein conservation (5 out of 7) was comparable with that of members of the *Herpesviridae *family and resembles the functional conservation of capsid structure in the *Herpesvirales *order.

### AngHV-1 envelope proteins

Removal of virus envelopes by treatment with a non-ionic detergent has allowed proteins to be assigned as components of the envelope versus capsid-tegument [16]. Proteins were principally defined as envelope proteins when present in the envelope fraction in higher concentrations (based on their emPAI) than in the capsid-tegument fraction, and when having certain characteristics of membrane proteins. A total of 30 proteins were identified by 1D gel/nanoLC-MS/MS in this sample and based on the former criteria 10 were annotated as putative envelope proteins (Table 2). Sixteen of the 20 non-envelope proteins were putative tegument proteins (Table 3), 4 were putative capsid proteins and little abundant. Eight of the envelope proteins comprised both a transmembrane domain and a signal peptide or anchor. The proteins encoded by ORF8 and ORF108 lacked a signal peptide, but were exclusively detected in the envelope fraction and not in the capsid-tegument fraction. The presumed multiple transmembrane protein encoded by AngHV-1 ORF49 was only detected in very low abundance in complete virion preparations and not in one of the virion fractions. The CyHV-3 homologue of this protein (ORF83) was not detected in CyHV-3 virions [17], possibly due to its low abundance. A signal peptide was predicted for only one other structural protein of AngHV-1 (encoded by ORF103), but not a transmembrane domain. Moreover, this protein was exclusively found in the capsid-tegument fraction, indicating that it is a putative tegument protein and not an envelope protein.

**Table 2:** Envelope proteins of AngHV-1 as identified by 1D gel/nanoLC-MS/MS in the envelope fraction

**ORF**^**a**^	NCBI ID	**Description**^**b**^	Predicted molecular mass (kDa)	**Number of peptides**^**c**^	Mascot score	**CyHV-3**^**d**^	Membrane protein type	**Trans-membrane domain(s)**^**e**^	Signal anchor or peptide	**N-glyco**^**f**^	**O-glyco**^**g**^
8	282174045		21.2	46	2390		Type 1	o1i	-	4	-
49^h^	282174086		26.4	-	-	ORF83	Type 3	i4i	Signal peptide	-	-
51	282174088	*Major envelope protein*	26.5	137	5970	*ORF81*	Type 3	i4i	Signal anchor	1	1
66	282174103		42.7	1	46		Type 1	o1i	Signal peptide	4	2
67	282174104	WBV spike protein/*Major glycoprotein*	152.9	54	2251	*ORF99*	Type 1	o1i	Signal peptide	13	8
71	282174108		11.4	29	1638		Type 1	o1i	Signal anchor	-	2
78	282174115		16.9	8	609		Type 3	i2i	Signal peptide	-	-
95	282174132	ISAV HA	41.6	190	11573		Type 1	o1i	Signal peptide	5	1
108	282174145	ORF80 family	108.2	4	183		Type 1	o1i	-	3	56
115	282174152		12.1	6	406		Type 1	o1i	Signal anchor	2	-
125	282174162	ORF109 family	120.5	2	189		Type 1	o1i	Signal peptide	16	16

^a^the ORF are ordered by number, ^b^properties newly suggested in this paper are presented in italics, ^c^number of peptides and Mascot score in the envelope fraction, ^d^homologous ORF in CyHV-3 are given, in case of marginal sequence identity (E-value > 10^-5 ^in a BLAST search limited to members of the *Alloherpesviridae *family) presented in italics, ^e^transmembrane domain(s) and orientation: o = outside, i = inside, number refers to number of transmembrane domains, ^f^predicted N-glycosylation site(s), ^g^predicted O-glycosylation site(s), and ^h^AngHV-1 ORF49 was only detected in very low abundance in complete virion preparations, and not in the envelope fraction.

**Table 3:** Tegument proteins of AngHV-1 as identified by 1D gel/nanoLC-MS/MS in the capsid-tegument and envelope fraction

**ORF**^**a**^	NCBI ID	**Description**^**b**^	Predicted molecular mass (kDa)	**Number of peptides (tegument)**^**c**^	Mascot score	**Number of peptides (envelope)**^**d**^	Mascot score	**CyHV-3**^**e**^	**Putative classification**^**f**^
14	282174051	ORF3 family	32.3	2	63	-	-		Tegument protein
16	282174053	ORF13 family	33.8	4	237	12	905		Tegument-associated protein
17	282174054	ORF13 family	25.8	13	851	29	1496		Tegument-associated protein
18	282174055		233.6	4	177	-	-	ORF42	Tegument protein
19	282174056		93.7	40	1717	7	372		Tegument protein
20	282174057		63.1	6	304	-	-	ORF45	Tegument protein
24	282174061	ORF13 family	29.3	12	842	39	2690		Tegument-associated protein
26	282174063		18.4	1	38	2	74		Tegument-associated protein
30	282174067		118.4	94	5311	70	3350	ORF97	Tegument-associated protein
32	282174069		20.0	2	101	5	192		Tegument-associated protein
34	282174071		195.0	176	9205	167	8820	ORF51	Tegument-associated protein
35	282174072		33.4	12	576	46	2113	ORF57	Tegument-associated protein
38	282174075		44.1	10	455	-	-	ORF70	Tegument protein
39	282174076		62.7	1	65	-	-	*ORF69*	Tegument protein
40	282174077		162.7	101	4570	21	921		Tegument protein
43	282174080		18.0	18	1444	35	2491		Tegument-associated protein
81	282174118		51.5	10	561	48	2673	ORF60	Tegument-associated protein
83	282174120	Cysteine protease domain/*Large tegument protein*	376.8	538	28887	298	16343	ORF62	Tegument-associated protein
103	282174140		25.0	5	273	-	-	ORF91	Tegument protein
114	282174151		20.7	19	913	61	3202		Tegument-associated protein
128	282174165		17.9	11	394	6	225		Tegument-associated protein
129	282174166		42.2	16	935	9	579		Tegument-associated protein

^a^the ORF are ordered by number, ^b^properties newly suggested in this paper are presented in italics, ^c^number of peptides and Mascot score in the capsid-tegument fraction, ^d^number of peptides and Mascot score in the envelope fraction, ^e^homologous ORF in CyHV-3 are given, in case of marginal sequence identity (E-value > 10^-5 ^in a BLAST search limited to members of the *Alloherpesviridae *family) presented in italics, and ^f^putative classification into a tegument or tegument-associated protein.

The two ORF encoding the most abundant envelope proteins demonstrated interesting sequence homologies. The most abundant AngHV-1 envelope protein is encoded by ORF51, which shows low sequence homology with CyHV-3 ORF81 (E-value = 10^-4^) in a directed search against members of the *Alloherpesviridae *family. CyHV-3 ORF81 encodes an abundant multiple transmembrane protein, thought to be the immunodominant envelope protein of CyHV-3 [42]. CyHV-3 ORF81 is the positional homologue of IcHV-1 ORF59, the latter being the major envelope protein [16]. While the homologue of this protein also seems to be a major envelope protein in AngHV-1, only a few peptides of this protein were found in CyHV-3 virions [17].

The second most abundant AngHV-1 envelope protein, encoded by ORF95, was previously shown to be related to the haemagglutinin-esterase protein of infectious salmon anaemia virus (ISAV) (E-value = 10^-16^) [15]. In the piscine orthomyxovirus ISAV this viral surface glycoprotein is responsible for viral attachment and release [43]. Herpesviruses are known to be capable of gene capture from cells or other viruses. Likewise AngHV-1 ORF95 might originate from ISAV or, for example, from a yet uncharacterized eel orthomyxovirus (Olga Haenen, personal communication).

AngHV-1 ORF67, encoding a large envelope protein, was previously shown to exhibit sequence homology with the *White bream virus *(WBV) spike protein (E-value = 10^-24^) [15], a protein probably mediating receptor binding and fusion between viral and cellular membranes [44]. A search for the yet unidentified AngHV-1 homologue of the presumed major glycoprotein present in other alloherpesviruses (IcHV-1 ORF46 [12], CyHV-3 ORF99 [14], RaHV-1 ORF46 [13] and RaHV-2 ORF72 [13]) resulted in low sequence homology of AngHV-1 ORF67 with RaHV-1 ORF46 (E-value = 10^-3^) in particular. The latter finding raises questions about the origin of AngHV-1 ORF67. One option might be that the current AngHV-1 ORF67 is the result of gene capture from WBV and subsequent genetic reassortment with the original major glycoprotein.

Human herpesviruses carry between 12 and 20 viral membrane proteins in their envelope [45]. For the alloherpesviruses CyHV-3 and AngHV-1, a total of 13 [17] and 11 (this paper) viral envelope proteins were predicted, respectively. Despite their large genomes, these alloherpesviruses seem to encode a relatively low number of membrane proteins. Many envelope proteins are specific to each herpesvirus type. However, among the members of the *Herpesviridae *family five glycoproteins are broadly conserved, namely gB, gH, gL, gM and gN [45]. Glycoprotein gB and a complex formed by gH and gL are involved in the fusion of the viral envelope and plasma membrane. Glycoproteins gM and gN form another complex. The fact that only two envelope proteins seem to be conserved among the members of the *Alloherpesviridae *family, is yet another indication that the evolutionary distance among fish and amphibian herpesviruses is greater than among mammalian, bird and reptile herpesviruses.

### AngHV-1 tegument proteins

Comparison of the proteins found in the capsid-tegument fraction with the proteins identified in purified capsids allowed the identification of the tegument proteins. Tegument proteins were defined as proteins found in the capsid-tegument fraction, but not (or only in trace amounts) in the purified capsid fraction, and not meeting the criteria as defined for envelope proteins. A total of 32 proteins were identified by 1D gel/nanoLC-MS/MS in the capsid-tegument sample, 22 of which were considered to be tegument proteins (Table 3). Six tegument proteins were exclusively found in the capsid-tegument fraction and for two other capsid-tegument proteins (ORF19 & ORF40) relatively high amounts were detected in the capsid-tegument fraction and relatively low amounts in the envelope fraction. Hence these proteins were called tegument proteins. The majority (14) of the proteins in the capsid-tegument fraction was, however, also found in the envelope fraction, indicating that these proteins are rather loosely bound to the tegument. These proteins were therefore classified as tegument-associated proteins. This distinction between true tegument and tegument-associated proteins is arbitrary and presumably depends on the fractionation conditions used. A more precise differentiation would be based on the interaction of the tegument proteins with either the capsid ("inner tegument") or the cytoplasmic domains of viral envelope proteins ("outer tegument") [40,46].

Nine sequence similarity based gene families have been identified within the AngHV-1 genome [15]. Most of these ORF encode proteins with unknown functions. Three of the four proteins of the ORF13 gene family were identified as tegument-associated proteins, indicating that this gene family encodes proteins with a tegument-related function.

Among the 22 tegument(-associated) proteins found, 10 showed sequence homology with CyHV-3 ORF, which therefore can now be classified as presumed tegument proteins. The largest AngHV-1 tegument-associated protein, ORF83, contains a cysteine protease domain in the N-terminal region homologous to the Ovarian Tumor gene in *Drosophila *[47]. This domain is also found in CyHV-3 ORF62 and IcHV-1 ORF65. Based on its size and conservation, this ORF has been suggested by Michel et al. [17] to encode the homologue of the large tegument protein UL36, which is conserved among the members of the *Herpesviridae *family [46,48]. UL36 is an essential and abundant structural polypeptide with multiple functions. It binds to the capsid on the one hand, and to several major tegument components on the other hand, suggesting that UL36 plays an important role in the structural organization of the tegument. A putative function could not be determined for any of the other AngHV-1 tegument(-associated) proteins.

Except for the large tegument protein, no significant sequence homology of tegument proteins was found with any of the other alloherpesviruses. This suggests that conservation of the tegument proteins is high between closely related alloherpesviruses, but hardly present throughout the whole family. This level of conservation reflects the greater divergence of the *Alloherpesviridae *family compared to the *Herpesviridae *family, among which almost a dozen tegument proteins are conserved [46,49,50]. More sequence data from other alloherpesviruses and functional characterization of yet identified tegument proteins is necessary to determine the exact level of tegument protein conservation [45].

### AngHV-1 virion composition

In order to unravel the relative abundance of the structural proteins of AngHV-1, proteins from purified virions separated by SDS-PAGE were excised from the gel in 20 serial slices. After in-gel digestion, the resulting peptides were analyzed by 1D gel/nanoLC-MS/MS. An in-solution trypsin digest MS approach, termed 2D nanoLC-MS/MS, was used as a complement to this gel based LC-MS/MS approach to enhance the recovery of peptides derived from proteins that are prone to aggregation, contain hydrophobic peptides, or are present at low abundance. The 1D gel and 2D nanoLC-MS/MS analyses of complete virions resulted in the identification of 33 and 27 viral proteins respectively, with an overlap of 26 proteins (Table 4). All but one of these proteins had already been identified in the virion fractions. AngHV-1 ORF49, presumably encoding a type 3 membrane protein, was detected in very low abundance in both complete virion analyses.

**Table 4:** Structural proteins of AngHV-1 as identified by 1D gel and 2D nanoLC-MS/MS

**ORF**^**a**^	NCBI ID	**Location**^**b**^	**Description**^**c**^	Predicted molecular mass (kDa)	**1D gel/nanoLC-MS/MS**^**d**^			**2D nanoLC-MS/MS**^**e**^		
					Number of peptides	Mascot score	EmPAI	Number of peptides	Mascot score	EmPAI
8	282174045	Envelope	*Membrane protein type 1*	21.2	29	1138	1.3	37	1339	2.59
*14*	282174051	Tegument	ORF3 family	32.3	-	-	-	-	-	-
16	282174053	Tegument-associated	ORF13 family	33.8	12	349	0.88	11	465	0.66
17	282174054	Tegument-associated	ORF13 family	25.8	21	1010	0.51	10	459	0.70
*18*	282174055	Tegument		233.6	-	-	-	-	-	-
19	282174056	Tegument		93.7	5	179	0.17	13	713	0.30
20	282174057	Tegument		63.1	3	123	0.19	-	-	-
24	282174061	Tegument-associated	ORF13 family	29.3	11	493	1.07	11	471	0.59
*26*	282174063	Tegument-associated		18.4	-	-	-	-	-	-
30	282174067	Tegument-associated		118.4	23	801	0.54	16	450	0.34
32	282174069	Tegument-associated		20.0	5	239	1.41	-	-	-
34	282174071	Tegument-associated		195.0	71	2641	1.32	58	2200	0.71
35	282174072	Tegument-associated		33.4	19	797	1.35	18	632	0.67
36	282174073	Capsid	Capsid triplex protein 2	40.2	29	1016	3.99	22	856	1.80
38	282174075	Tegument		44.1	4	142	0.39	-	-	-
39	282174076	Tegument		62.7	2	57	0.12	-	-	-
40	282174077	Tegument		162.7	22	783	0.56	13	446	0.29
42	282174079	Capsid	*Capsid triplex protein 1*	42.9	20	979	1.73	17	558	1.42
43	282174080	Tegument-associated		18.0	4	121	0.48	8	291	1.12
48	282174085	Capsid		102.6	32	1112	1.18	26	876	0.97
49	282174086	Envelope^f^	*Membrane protein type 3*	26.4	1	38	0.14	1	42	0.14
51	282174088	Envelope	*Major envelope protein/Membrane protein type 3*	26.5	30	1042	4.70	30	972	2.18
57	282174094	Capsid	Capsid protease-and-scaffolding protein	78.0	9	345	0.45	8	328	0.43
66	282174103	Envelope	*Membrane protein type 1*	42.7	6	458	0.18	-	-	-
67	282174104	Envelope	WBV spike protein/*Major glycoprotein*/*Membrane protein type 1*	152.9	25	948	0.43	15	577	0.26
71	282174108	Envelope	*Membrane protein type 1*	11.4	4	172	0.82	3	103	0.33
78	282174115	Envelope	*Membrane protein type 3*	16.9	4	146	0.23	-	-	-
81	282174118	Tegument-associated		51.5	7	226	0.63	1	56	0.07
83	282174120	Tegument-associated	Cysteine protease domain/*Large tegument protein*	376.8	166	6652	1.46	104	3870	0.71
95	282174132	Envelope	ISAV HA/*Membrane protein type 1*	41.6	54	2069	1.81	52	2256	1.48
100	282174137	Capsid		61.1	2	66	0.13	1	33	0.03
*103*	282174140	Tegument			-	-	-	-	-	-
104	282174141	Capsid	Major capsid protein	139.9	213	8402	5.33	104	4058	2.56
108	282174145	Envelope	ORF80 family	108.2	-	-	-	1	33	0.06
114	282174151	Tegument-associated		20.7	28	852	3.64	13	550	1.27
*115*	282174152	Envelope			-	-	-	-	-	-
*125*	282174162	Envelope	ORF109 family		-	-	-	-	-	-
126	282174163	Capsid		22.4	1	30	0.17	-	-	-
128	282174165	Tegument-associated		17.9	4	139	0.48	1	60	0.21
129	282174166	Tegument-associated		42.2	8	290	0.19	6	238	0.18

^a^the ORF are ordered by number with the ORF only detected in the virion fractions presented in italics, ^b^location as determined in this paper, ^c^properties newly suggested in this paper are presented in italics, ^d^number of peptides, Mascot score and emPAI in the complete virion fraction as determined by 1D gel/nanoLC-MS/MS, ^e^number of peptides, Mascot score and emPAI in the complete virion fraction as determined by 2D nanoLC-MS/MS, ^f^AngHV-1 ORF49 was not detected in the envelope fraction and classified as an envelope protein only on the basis of predicted structural properties.

**Figure 2:**
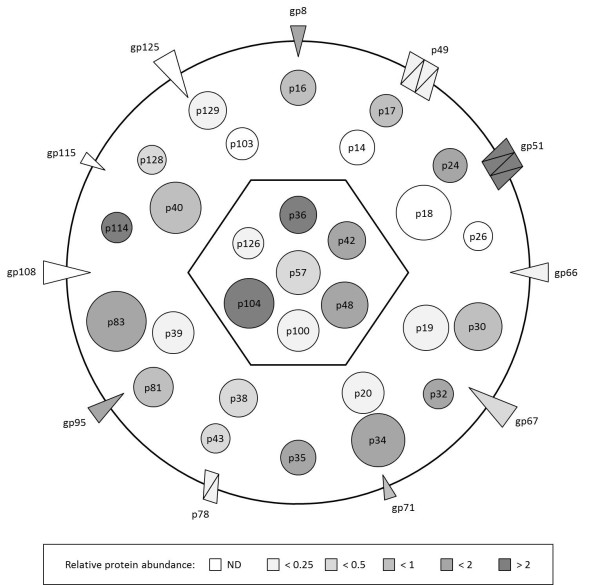
**Schematic representation of the protein composition of mature extracellular AngHV-1 virions**. The typical herpesvirus compartments are indicated as a hexagon (capsid), circle (envelope) and the space in between (tegument). The location of the structural proteins is indicated. Proteins identified as true tegument proteins directly surround the capsid, proteins identified as tegument-associated proteins are located in the outer part of the tegument. The orientation of the envelope proteins indicates the number of transmembrane domains. The predicted protein mass is logarithmically indicated in size. The relative abundance (emPAI) as determined for the 1D gel/nanoLC-MS/MS analyses of complete virions is indicated in color intensity (see scale).

For the 1D gel and 2D nanoLC-MS/MS analyses of complete virions the emPAI was calculated to estimate protein relative abundance (Table 4). The emPAI from both analyses is generally comparable. A schematic representation of the AngHV-1 virion was made based on predicted protein mass, localization of the structural proteins of AngHV-1 as determined by analyses of the respective fractions, and the relative abundance in complete virions as determined for the protein extract analyzed by 1D gel/nanoLC-MS/MS (Figure 2).

### Host proteins associated with AngHV-1 virions

Several cellular host proteins end up in mature herpesvirus virions, either intentionally or accidentally in the process of virus assembly and release from the cell. We also performed searches for non-viral host-originating proteins for all LC-MS/MS datasets acquired in this study. The proteins were identified by searching the peptides against a bony vertebrate database, since there are only very few genomic *Anguilla *spp. sequences available. In the 1D gel and 2D nanoLC-MS/MS analyses of complete virions, 30 and 15 host proteins were detected, respectively, with an overlap of 3 proteins (Additional file 1). A total of 28 unique host proteins associated with AngHV-1 virions were found, compensating for the several hits against protein homologues in different (fish) species. This number is somewhat higher than the number of host proteins found to be associated with CyHV-3 [17]. It resembles numbers found for several mammalian herpesviruses, either cushion [23,25] or gradient purified [24], however.

The host proteins found to be associated with AngHV-1 virions include cytoskeleton proteins (α-actin, β-actin, actin-depolymerization factor, filamin, keratin, profilin, septin, etc.), proteins involved in transport (fatty acid binding proteins, lipocalin, myelin), an adrenoreceptor-like protein, proteins involved in glycolysis (aldolase and glyceraldehyde-3-phosphate dehydrogenase) and protein glycosylation, regulatory proteins (ubiquitin and a WD repeat containing protein), a protein involved in translation control (Sp5 transcription factor), proteins involved in the immunological response (pentraxin) and stress-response (heat shock proteins 70 & 90), and several proteins with yet unknown functions.

Several of the classifiable virion associated host proteins have also been described in CyHV-3 and mammalian herpesviruses [16-20,22-25]. The composition of the set of incorporated host proteins might, however, be influenced by the type of cell culture used for virus propagation. All virion associated host proteins were found in low concentrations of only 1 to a maximum of 18 peptides per protein (Additional file 1), suggesting that these proteins are only minor components of the virion. Although many of the identified host proteins have previously been associated with herpesvirus virions, it is possible that some of these proteins represent minor cellular contaminants of the virion preparations.

### Evaluation of the approach

In this study, three approaches were followed for the detection of the structural proteins of AngHV-1. The first and most straight-forward method was the in-solution trypsin digest MS approach termed 2D nanoLC-MS/MS. A total of 27 unique structural proteins were identified. The second approach was the extraction and separation of AngHV-1 virion proteins by SDS-PAGE, followed by excision of 20 contiguous sections of the gel along the migration path, in-gel trypsin digestion and subsequent nanoLC-MS/MS analysis. This approach resulted in the identification of another 7 unique AngHV-1 structural proteins. The third approach, which intended to assign the identified proteins to the different AngHV-1 virion compartments, resulted in the identification of another 6 structural proteins. The proteins detected by only one of the approaches were generally little abundant.

A total of 40 structural proteins of AngHV-1 were identified (Tables 1, 2, 3 and 4). This number is not likely to represent the actual total number of AngHV-1 structural proteins, but will nevertheless represent the majority. For the related CyHV-3 virion, a similar number of structural proteins was recently identified, but Michel et al. used several additional protein extraction methods before separating the virions by SDS-PAGE [17]. Based on their results we decided to use the two most efficient extraction and separation procedures for the current study, namely GC extraction followed by in-solution trypsin digestion for the 2D nanoLC-MS/MS approach, and protein extraction with SDS followed by separation by SDS-PAGE and in-gel trypsin digestion for the 1D gel/nanoLC-MS/MS. Higher virion concentrations as well as additional analyses of separate virion fractions significantly contributed to the recovery of peptides derived from proteins present in low abundance.

When Davison and Davison identified the structural proteins of IcHV-1 in 1995, they found a total of 16 principal structural proteins [16]. We followed their capsid retrieval and virion fractionation techniques, confirmed by EM and SDS-PAGE, but used a more sensitive MS technique. The number of structural proteins found for AngHV-1 was significantly higher than the number found earlier for IcHV-1. Generally, the capsid retrieval and virion fractionation techniques worked well in combination with LC-MS/MS. Nevertheless, several highly abundant proteins showed some overspill in fractions where the proteins were not expected to be. This probably indicates that the virion fractionation techniques were not conclusive. In addition, it could be possible that proteins from which only one or two peptides were found are actually contaminating high abundant non-structural proteins, detected by the highly sensitive LC-MS/MS technique used.

Several observations support the accuracy of the final results of our approach. First, the number of proteins found in the different fractions was highly comparable to those determined by recent comprehensive characterization of extracellular herpes simplex virus type 1 and pseudorabies virus virions [23,25]. Second, all AngHV-1 sequence similarity based homologues of CyHV-3 structural proteins were found in AngHV-1 virions. Third, the proteins with a sequence predicted function were found in the expected fractions. Fourth, as for the low abundant proteins, several of these proteins have homologues in CyHV-3 and were accordingly detected in CyHV-3, which were purified using a different protocol [17]. Fifth, all proteins identified as envelope proteins showed many if not all of the basic characteristics of an envelope protein, whilst none of the other structural proteins showed a predicted transmembrane domain, and a signal peptide was predicted for only one non-envelope protein. Sixth, non-structural proteins such as proteins involved in DNA replication, DNA packaging or presumably secreted immunomodulatory proteins were not identified.

## Conclusions

The identification and localization of the structural proteins of AngHV-1 in this study adds to the fundamental knowledge of members of the *Alloherpesviridae *family, especially for the known members of the related *Cyprinivirus *genus. The localization and putative function of the identified AngHV-1 structural proteins can now be extrapolated for homologous genes in other alloherpersviruses. In addition, the results presented give a first indication of the conservation of structural proteins within the *Alloherpesviridae *family. Conservation is high in the capsid fraction, limited in the tegument fraction and low in the envelope fraction. In this respect the *Alloherpesviridae *family resembles the *Herpesviridae *family, yet the evolutionary distance among fish and amphibian herpesviruses is significantly greater than among mammalian, bird and reptile herpesviruses. For AngHV-1 in particular, the results of this study will facilitate more directed functional characterization of proteins of interest. Moreover, this information is essential in further studies on the pathobiology of this virus, and will support the development of specific diagnostic tools and vaccines.

## Competing interests

The authors declare that they have no competing interests.

## Authors' contributions

SJvB designed the study, carried out the virus culture, virion purification & fractionation, capsid isolation, SDS-PAGE, sample preparations for LC-MS/MS, bioinformatic analyses and drafted the manuscript. BL performed the 1D gel/nanoLC-MS/MS and 2D nanoLC-MS/MS measurements of complete virions, capsids and virion (including sample preparations), supervised by RW and coordinated by AV. OLMH headed the cell and virus cultures. SB assisted in the sample preparations for LC-MS/MS and performed the LC-MS/MS analyses of the U capsids, supervised by JJMV. BPHP, PJMR and AV advised on the study design and manuscript. MYE coordinated the study. All authors read and approved the final manuscript.

## Supplementary Material

Additional file 1 **Table S1: Host proteins associated with AngHV-1 virions as identified by 1D gel/nanoLC-MS/MS and 2D nanoLC-MS/MS**. This table contains the non-viral host-originating proteins identified in the 1D gel and 2D nanoLC-MS/MS analyses of complete virions by searching the peptides against a bony vertebrate database. Thirty and 15 host proteins were detected, respectively, with an overlap of 3 proteins, representing a total of 28 unique host proteins associated with AngHV-1 virions. Click here for file
