# Supplementary material for: Identification and localization of the structural proteins of anguillid herpesvirus 1
Source: Vet Res. 2011 Oct 5;42(1):105. doi: 10.1186/1297-9716-42-105 (PMC3203048; doi:10.1186/1297-9716-42-105)
Supplement: Additional file 1 — Table S1: Host proteins associated with AngHV-1 virions as identified by 1D gel/nanoLC-MS/MS and 2D nanoLC-MS/MS. This table contains the non-viral host-originating proteins identified in the 1D gel and 2D nanoLC-MS/MS analyses of complete virions by searching the peptides against a bony vertebrate database. Thirty and 15 host proteins were detected, respectively, with an overlap of 3 proteins, representing a total of 28 unique host proteins associated with AngHV-1 virions. [file 1297-9716-42-105-S1.DOC]

### Additional file 1: Host proteins associated with AngHV-1 virions as identified by 1D gel/nanoLC-MS/MS and 2D nanoLC-MS/MS.

| **Category^a^** | **Host protein^b^** | **Species origin^c^** | **NCBI ID** | **Predicted molecular mass (kDa)** | **1D gel/nanoLC-MS/MS** | | **2D nano LC-MS/MS** | |
| --- | --- | --- | --- | --- | --- | --- | --- | --- |
|  |  |  |  |  | **Number of peptides^d^** | **Mascot score** | **Number of peptides^e^** | **Mascot score** |
| Cytoskeleton | Actin clone 403 | *Artemia sp.* | 113255 | 42.2 | 18 | 431 | - | - |
|  | Actin depolymerisation factor | *Tetraodon nigroviridis* | 47225287 | 18.9 | 1 | 69 | - | - |
|  | Alpha-actin | *Salmo trutta* | 8489855 | 42.2 | - | - | 10 | 292 |
|  | Beta-actin | *Anguilla anguilla* | 82798416 | 17.8 | 10 | 325 | - | - |
|  | “ | *Phoxinus oxycephalus* | 7546805 | 42.0 | - | - | 14 | 406 |
|  | “ | *Pungitius pungitius* | 66731680 | 40.5 | - | - | 14 | 405 |
|  | “ | *Rhodeus notatus* | 10442729 | 42.0 | 18 | 446 | - | - |
|  | Cytoskeleton-associated protein 5-like | *Danio rerio* | 326680017 | 93.8 | 2 | 44 | - | - |
|  | Filamin A | *Danio rerio* | 189535920 | 271.6 | 5 | 137 | 1 | 55 |
|  | “ | *Ictalurus punctatus* | 90103410 | 9.6 | 1 | 111 | - | - |
|  | Profilin-2 | *Ictalurus furcatus* | 308321486 | 13.9 | 1 | 73 | - | - |
|  | Sept2 protein | *Danrio rerio* | 32766415 | 39.2 | 1 | 57 | - | - |
|  | Simple type II keratin K8a (S1) | *Oncorhynchus mykiss* | 185132941 | 59.2 | 6 | 139 | - | - |
|  | Slow myotomal muscle tropomyosin | *Salmo trutta* | 3063940 | 32.7 | - | - | 1 | 45 |
| Glycolysis | Aldolase | *Ictalurus punctatus* | 27883578 | 17.4 | 1 | 48 | - | - |
|  | Glyceraldehyde-3-phosphate dehydrogenase | *Anguilla japonica* | 10567305 | 32.1 | 1 | 61 | - | - |
| Immunological response | Pentraxin | *Gasterosteus aculeatus* | 194277689 | 23.7 | 12 | 93 | 2 | 44 |
| Protein glycosylation | Alpha-N-acetylgalactosamine alpha-2,6-sialyltransferase | *Takifugu rubripes* | 5002573 | 27.3 | - | - | 5 | 46 |
| Receptor-like proteins | Alpha-1A adrenoceptor-like | *Danio rerio* | 125823286 | 53.7 | - | - | 2 | 45 |
| Regulatory proteins | Ubiquitin | *Salmo sp.* | 223061 | 8.5 | - | - | 1 | 49 |
|  | WD repeat domain 1 | *Danio rerio* | 37595360 | 67.1 | 1 | 62 | - | - |
| Stress response | Heat shock protein 70 | *Carassius gibelio* | 28569550 | 71.4 | 11 | 392 | - | - |
|  | “ | *Ctenopharyngodon idella* | 323146387 | 71.7 | - | - | 5 | 144 |
|  | “ | *Danio rerio* | 153792281 | 70.9 | 6 | 256 | 4 | 131 |
|  | “ | *Danio rerio* | 1865782 | 71.5 | 10 | 345 | - | - |
|  | “ | *Oncorhynchus mykiss* | 17129570 | 71.3 | 8 | 263 | - | - |
|  | “ | *Oreochromis mossambicus* | 3004463 | 70.5 | 12 | 324 | - | - |
|  | “ | *Pelteobagrus fulvidraco* | 237688438 | 71.0 | 10 | 348 | - | - |
|  | “ | *Rachycentron canadum* | 62529292 | 24.0 | 5 | 133 | - | - |
|  | “ | *Scophthalmus maximus* | 144952758 | 71.4 | 10 | 322 | - | - |
|  | “ | *Silurus meridionalis* | 126116091 | 71.1 | 11 | 345 | - | - |
|  | “ | *Takifugu rubripes* | 1620388 | 70.3 | 8 | 264 | - | - |
|  | Heat shock protein 90 | *Oncorhynchus tshawytscha* | 1899173 | 83.9 | 1 | 93 | - | - |
| Transcription-translation control | Sp5 transcription factor-like | *Danio rerio* | 35902791 | 40.4 | 6 | 43 | - | - |
| Transport proteins | Fatty acid binding protein H8-isoform | *Chaenocephalus aceratus* | 2738182 | 15.0 | - | - | 2 | 104 |
|  | Fatty-acid binding protein heart-type | *Cyprinus carpio* | 281333450 | 14.7 | - | - | 2 | 87 |
|  | Lipocalin | *Tetraodon nigroviridis* | 47222259 | 13.4 | 2 | 68 | - | - |
|  | Myelin P2 protein | *Osmerus mordax* | 225708474 | 15.3 | 1 | 69 | - | - |
| Unknown function | Unnamed protein product | *Tetraodon nigroviridis* | 47204907 | 15.9 | - | - | 3 | 47 |
|  | Unnamed protein product | *Tetraodon nigroviridis* | 47222608 | 25.3 | - | - | 1 | 42 |
|  | Unnamed protein product | *Tetraodon nigroviridis* | 47210777 | 20.2 | 2 | 52 | - | - |
|  | Zgc:109744 | *Danio rerio* | 71534073 | 88.7 | 2 | 45 | - | - |

^a^the host proteins are classified by category and alphabetically ordered, ^b^Homologous protein in different host species are indicated by “, ^c^as a complete *A. anguilla* sequence was not available, most homologies were found in other species, ^d^number of peptides and Mascot score in the complete virion fraction as determined by 1D gel/nanoLC-MS/MS, ^e^number of peptides and Mascot score in the complete virion fraction as determined by 2D nanoLC-MS/MS
